# Participatory geographic mapping and activity space diaries: innovative data collection methods for understanding environmental risk exposures among female sex workers in a low-to middle-income country

**DOI:** 10.1186/s12942-021-00279-9

**Published:** 2021-05-31

**Authors:** Erica Felker-Kantor, Caluz Polanco, Martha Perez, Yeycy Donastorg, Katherine Andrinopoulos, Carl Kendall, Deanna Kerrigan, Katherine P. Theall

**Affiliations:** 1 Department of Global Community Health and Behavioral Sciences, Tulane School of Public Health and Tropical Medicine, 1440 Canal Street, New Orleans, LA 70112 USA; 2 La Universidad Autónoma de Santo Domingo, Santo Domingo, Dominican Republic; 3 Instituto Dermatológico Dominicano Y Cirugía de Piel, Santo Domingo, Dominican Republic; 4 Federal University of Ceará, School of Medicine, Program in Graduate Studies in Community Health, Fortaleza, Brazil; 5 Department of Prevention and Community Health, George Washington University, Washington, DC USA

**Keywords:** Activity space mapping, Participatory geographic mapping, Risk environments, Low-to middle-income countries, HIV

## Abstract

**Background:**

A common approach for measuring place-based exposure is to use geographically-defined administrative boundaries and to link neighborhood characteristics at this level. This approach, however, may not be feasible in low-to middle-income countries where neighborhood-level data are limited or unavailable, and administrative boundaries are often unstandardized and not proportional to population size. Furthermore, such traditional approaches may not be appropriate for marginalized populations whose environments can be more difficult to study. In this paper, we describe two innovative and feasible methods to generate geospatial data to characterize and assess the role of risk environments on drug use among female sex workers living with HIV in the Dominican Republic.

**Methods:**

Participatory geographic mapping and daily activity space travel diaries were employed.

**Results:**

The methods presented in this study were feasible to implement, acceptable by study participants, and yielded rich geospatial data to analyze the impact of contextual factors on risk behaviors of female sex workers in a low-to middle-income country.

**Conclusion:**

Participatory geographic mapping and activity space diaries are two alternative methods for collecting geospatial data among hard-to-reach populations in resource constrained settings. Moreover, the methods are interactive and educational, allowing study participants to take an active role in the data collection process and potentially allowing for a deeper understanding of place-based effects on health and behavior.

## Background

Efforts to understand HIV risk are increasingly focused on broader, contextual factors that shape individual behavior. One aspect of this shift toward a socio-ecological understanding of disease is the recognition that *place* may exert substantial influence on individual psychological and physical health. According to Rhodes et al., “the risk environment is the most important determinant of HIV transmission and prevention” [1]. The risk environment framework views risk behavior as a product of the environment and social experiences in which individuals participate [2]. Documenting and understanding the effect that environments have on risk behaviors is important for the development of multilevel interventions that address the structural determinants of health.

Researchers have used tools from a variety of disciplines including geography, sociology, and economics to characterize the social and physical characteristics of risk environments and assess relationships with health behaviors and outcomes. Methods from spatial epidemiology and the field of neighborhood effects research have been particularly popular given the range of valid and reliable tools and increasing access to geographic information systems (GIS) that help facilitate analysis of location-based data [3]. In high-income settings, a common approach for deriving place-based exposure measures is to use a set of geographically-defined administrative boundaries (e.g., census tracts, zip codes) according to neighborhood of residence and to aggregate neighborhood characteristics (e.g., percentage of households below the poverty line, number of fast food outlets, rate of crime events) to the level of the administrative boundary [4–6].

One concern with this approach, however, is that study results are often inconsistent due to the modifiable areal unit problem (MAUP) [7]. MAUP is a source of statistical bias that occurs when working with spatial data. MAUP arises when arbitrarily defined geographic areas are used for measurement and reporting of spatial phenomena. MAUP demonstrates that analytical differences may occur depending on the size of the geographic units (the scale effect) and how the configuration of study area is divided (the zoning effect) [8]. Furthermore, defining exposure according to residential location ignores exposure to places beyond residential areas. This problem, known as the uncertain geographic context problem (UGCoP), arises because of spatial uncertainty of the actual areas that have contextual influence on individuals and the temporal uncertainty in duration of exposure [9]. Ultimately, both MAUP and UGCoP may lead to inaccuracies in measures of exposure, spatial misclassification, and spurious findings [10].

In many low-to middle-income countries (LMIC) neighborhood-level data are limited or unavailable, and administrative boundaries may be unstandardized and not proportional to population size due to the rapid growth of urban cities and inconsistent numbering of street addresses. This can pose a challenge for researchers interested in measuring risk environments as predictors or effect modifiers of health outcomes in LMIC settings. Furthermore, risk environments are often studied in relation to HIV risk behaviors of key populations (e.g., female sex workers (FSWs), men who have sex with men (MSM), and injection drug users (IDUs)), but given the pervasive stigma, vulnerability, criminalization and high mobility of these populations, methodological approaches that derive place-based exposures based on a set of geographically-defined administrative boundaries are not feasible or appropriate [11]. To help facilitate risk environment research among key populations in LMIC, more methods-based studies that describe the development, implementation, and feasibility of tools used to measure risk environment exposure are needed.

The purpose of this paper is to describe the methods used in a pilot study that aimed to characterize the risk environments of FSWs living with HIV in the Dominican Republic and to assess the relationship between risk environment exposure and daily drug use. The methods used to capture and measure FSW risk environments were based on approaches from the field of neighborhood effects research but adapted for the study population and setting. The methods included: participatory geographic mapping and activity space mapping.

### Participatory geographic mapping

Participatory geographic mapping is the process of gathering geographic and spatial data through an interactive human process using integrated methods and technologies. The idea behind participatory geographic mapping is to bring the practices of GIS to the local level to gather information while also promoting knowledge production and empowerment through participation [12]. It involves the creation of spatial information and geocoded knowledge to be used for spatial decision-making and is directly related to enhancing the community’s understanding of place and developing awareness about their surroundings [13]. Participatory geographic mapping has emerged as a valuable tool for collecting spatial data in LMICs where there is limited access to geographic data and it has been successfully used in previous studies with hard-to-reach populations including FSWs, IDUs, and MSM [5, 14–16].

### Activity space mapping

In recent years, the concept of ‘activity space’, coupled with the availability of real-time geographic positioning system (GPS) tracking technologies, has emerged as a more accurate and objective approach to measuring place-based exposure compared to using fixed administrative boundaries and residential location. Activity space is defined as “the local areas within which individuals habitually move about in the course of their daily activities” [10]. Activity space research examines all spaces—whether physical or social—in which daily activities occur [17]. The examination of activity spaces provides for more precise operationalized measures that capture the complexities of human spatial behavior and all the accompanying psychological, social, and health-related experiences within those spaces. The majority of activity space studies in public health have focused on the feasibility of using real-time GPS devices to measure daily mobility patterns [17, 18]. Among the few studies that have compared contextual risks in activity spaces to residential areas, significant differences in exposure levels have been detected [18–21].

## Methods

### Setting

The Dominican Republic is one of the largest sex tourism destinations in the Caribbean with an estimated 100,000 women involved in the sex industry [22]. Sex work is not explicitly illegal in the country for people over the age of 18. Historically, the majority of sex work was establishment-based, but recent estimates suggest that more than 60% of FSWs independently solicit clients from streets, parks, beaches or other public places. FSWs who are establishment-based tend to work in brothels, bars, discos, liquor stores, or car washes. Even though sex work is not illegal in the country, harassment by police and other law enforcement officials is common [23, 24].

### Study description

The pilot study employed a micro-longitudinal observational study design and was nested within an ongoing 5 year (2016–2021) NIH-funded parent study (5R01MH110158) in Santo Domingo [25]. Further details on the parent study are described elsewhere (see [25]). Women were eligible to participate in the pilot study if they met all the parent study’s inclusion criteria which included being at least 18 years of age, having a confirmed HIV positive diagnosis determined by a single rapid test, and having exchanged sex for money in the month prior to study enrollment. Additional inclusion criteria required for the pilot study included that women had used drugs in the 6 months prior to data collection [required for half the sample], were willing and able to complete a paper-based travel diary for 7 days, and were willing and able to answer electronic daily behavior diary questions for 7 days.

Participants were recruited from the parent study using selective/purposive sampling based on drug use. To determine drug use status, results from the parent study baseline survey were analyzed to determine participants who ever used/used drugs in the 6 months prior to data collection. Among the 200 women in the parent study at baseline, 36.5% had ever used drugs and 16% were current drug users. Thus, for the current study, drug using participants were randomly sampled from the 16% of current drug users in the parent study. The goal was to enroll a minimum of 25 drug using participants.

Non-drug using participants were randomly selected based on viral load detectability as defined by the parent study. Non-drug users were categorized as viral load detectable and non-detectable, and every 5th participant from each group was selected as a potential participant. The goal was to enroll a minimum of 25 non-drug using participants, half with detectable viral loads and half non-detectable. Adhering to the parent study’s recruitment processes, participant contact information was obtained from the IDCP coordinator and FSW peer navigators were used to contact and recruit participants.

**Fig. 1:**
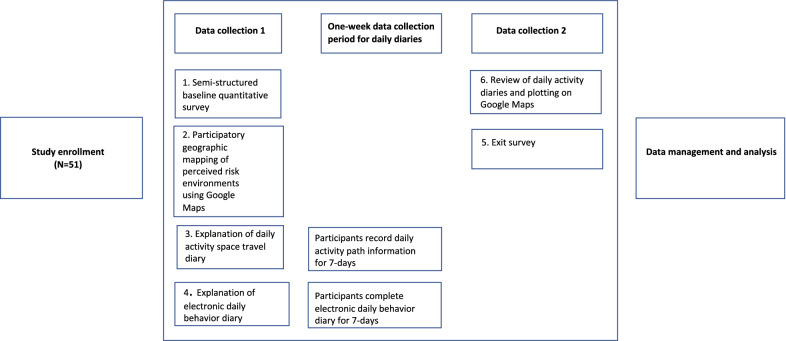
Diagram of the research process

Data collection activities included: (1) baseline questionnaire; (2) participatory geographic mapping; (3) daily activity space travel diary collected for 7 days and; (4) daily behavior diary collected for 7 days. Data collection instruments and measures were piloted, translated to Spanish, and adapted to the Dominican context. Written informed consent was obtained from all participants. Study enrollment was held at the *Instituto Dermatológico Dominicano y Cirugía de Piel (IDCP*) in Santo Domingo where the parent study was located. Ethics approval from the Internal Review Boards (IRB) at Tulane University and IDCP was obtained. The final analytic sample size was N = 51. A diagram of the data collection process is displayed in Fig. 1.

Participants received $10 USD for participating in the study and an additional $3 per day for completion of the activity space travel diary and the daily behavior diary (1 travel diary a day, 1 behavior diary a day × 7 days = $21). Compensation was provided at time of submission of the activity space travel diary following the 7-day data collection period. Transportation to and from the study site was covered for 2 trips. At the time of enrollment, cellphones were loaded with a pre-paid 7-day data package to cover Internet costs for the daily behavior diary.

### Participatory geographic mapping of perceived risk environments

Given the limited availability of neighborhood-level data in the Dominican Republic, we used participatory geographic mapping to obtain data on perceived neighborhood risk characteristics and locations in Santo Domingo [26]. During baseline data collection, participants were asked to identify locations or areas they perceived as unsafe. More specifically, using Google Maps, we asked participants to locate areas and locations for sex work, crime and violence, police presence, drug use and trafficking, and poverty. While sex work itself is not inherently risky it becomes risky due to violence, stigma, lack of legal protection, forced substance use, and seclusion, which is why locations of sex work were characterized as potential risk environments [27]. Similarly, while poverty may not cause violence, areas with higher poverty rates are disproportionately affected by crime and violence which is why areas with higher levels of poverty were included as possible perceived risk environments [28]. Participants were then asked to rate the perceived riskiness or level of unsafety of the location as ‘high’, ‘medium’ or ‘low’. For each location participants were asked to provide the address or nearest cross section and a temporary point was placed at the location. Google Street View was used to verify locations and geographic coordinates (latitude and longitude) were obtained. For locations that were polygon or area-based, spatial boundaries were identified using cross-streets and landmarks. Using the satellite imagery as a guide, boundaries were demarcated and digitized. The name of each perceived risk environment, risk characteristic (e.g., sex work, crime and violence, police presence, drug use and trafficking, and poverty), risk rating (e.g., high, medium, low), and geographic coordinates were recorded in an Excel file. This exercise took an average of 30 min per participant.

Aggregated information per perceived risk environment location were calculated, including the number of times the location was mentioned by participants, the number of descriptive risk characteristics assigned to the location, and an average risk rating. The average risk rating was calculated by assigning a value to the risk category (where high risk = 3, medium risk = 2, and low risk = 1), and summing the total risk score across participants divided by the number of times mentioned. A weighted risk rating was also calculated taking into consideration the number of risk characteristics assigned to the location/area. The data were de-duplicated and imported into ArcGIS 10.6 (ESRI, Redlands, CA) where they were joined and overlaid on a base map of Dominican census tracts (*barrio parajes*) and road data.

### Daily activity space travel diary

The best practice for activity space mapping is to use GPS technology because it minimizes recall and respondent bias and requires minimal investment by the participant; location, time, and speed are recorded in real-time at pre-determined time intervals (e.g., every minute). However, considering our study population and the context of sex work, the local research team was hesitant to use GPS for issues related to privacy and vulnerability. In the Dominican Republic, sex work is not explicitly illegal for people over the age of 18, but sex workers are frequently subjected to harassment by police and other law enforcement officials, violence by clients and partners, and societal stigma, so data are very sensitive. As an alternative to the more invasive form of GPS tracking, we captured participant activity paths for 7 days using a paper-based travel diary adapted from a study by Kwan et al. [5]. This format provided participants the flexibility to complete the diaries when they were in a secure location without the risk of others bothering them or finding out sensitive information.

**Fig. 2:**
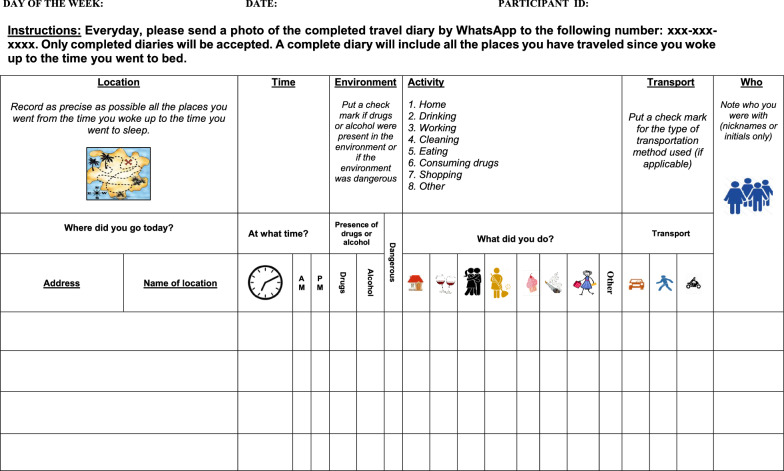
Example of the paper-based travel diary to collect daily activity space data

During study enrollment participants received 7 travel diary forms, one for each day of the week. Participants were asked to record the location, address, time, presence of drugs and alcohol in the environment, main activity, transport method, and whom they were with for each place visited during the day from the time they woke up until they went to bed. As depicted in Fig. 2, the diary was designed as a grid with columns and rows. Visual icons accompanied by simple instructions were used to indicate the information to be recorded. Participants were showed how to complete the diary and provided the PI’s contact information in case they had questions.

To verify daily completion of travel diaries, participants were asked to send the PI a daily snapshot photo of the completed travel diary labeled with the participant’s unique ID via WhatsApp. Participants received a daily reminder via WhatsApp to complete the diary and to send a photo of the completed travel diary by the following morning by 12:00 PM, which was selected as the submission time due to the nature of the participants work and late hours. The information from the daily travel diaries was input to an electronic version of the travel diary. At the end of the 7-day data collection period, participants returned the paper travel diaries and were asked to clarify any entries that lacked information that was necessary for recording the latitude and longitude of each location. With the help of the participants, each location visited during the week was plotted on a Google Map file and the latitude and longitude recorded in an electronic version of the travel diary for that participant.

Each participant’s weekly travel diary information, which included latitude and longitude coordinates for each location visited during the week, was imported into ArcGIS 10.6 (ESRI, Redlands, CA). Activity paths were generated using the shortest roadway network tool, connecting point locations in order by day and time using the shortest distance along the roadway network. To calculate activity space risk exposure measures, activity paths were overlaid on the base map of Santo Domingo joined with the risk environment data and additional secondary spatial point data of risk outlets from the 2014 PLACE study [29]. The 2014 PLACE study was conducted in 6 regions of the country known to have high HIV prevalence. One objective of the study was to characterize and map risk locations frequented by key populations (e.g., sex workers, MSM, IDUs etc.) such as car washes, liquor stores, bars, hotels, construction areas, nightclubs, brothels etc. Locations were identified and captured via interviews with community informants about where key populations socialize and meet sexual partners.

## Results

### Participatory geographic mapping of perceived risk environments

**Fig. 3:**
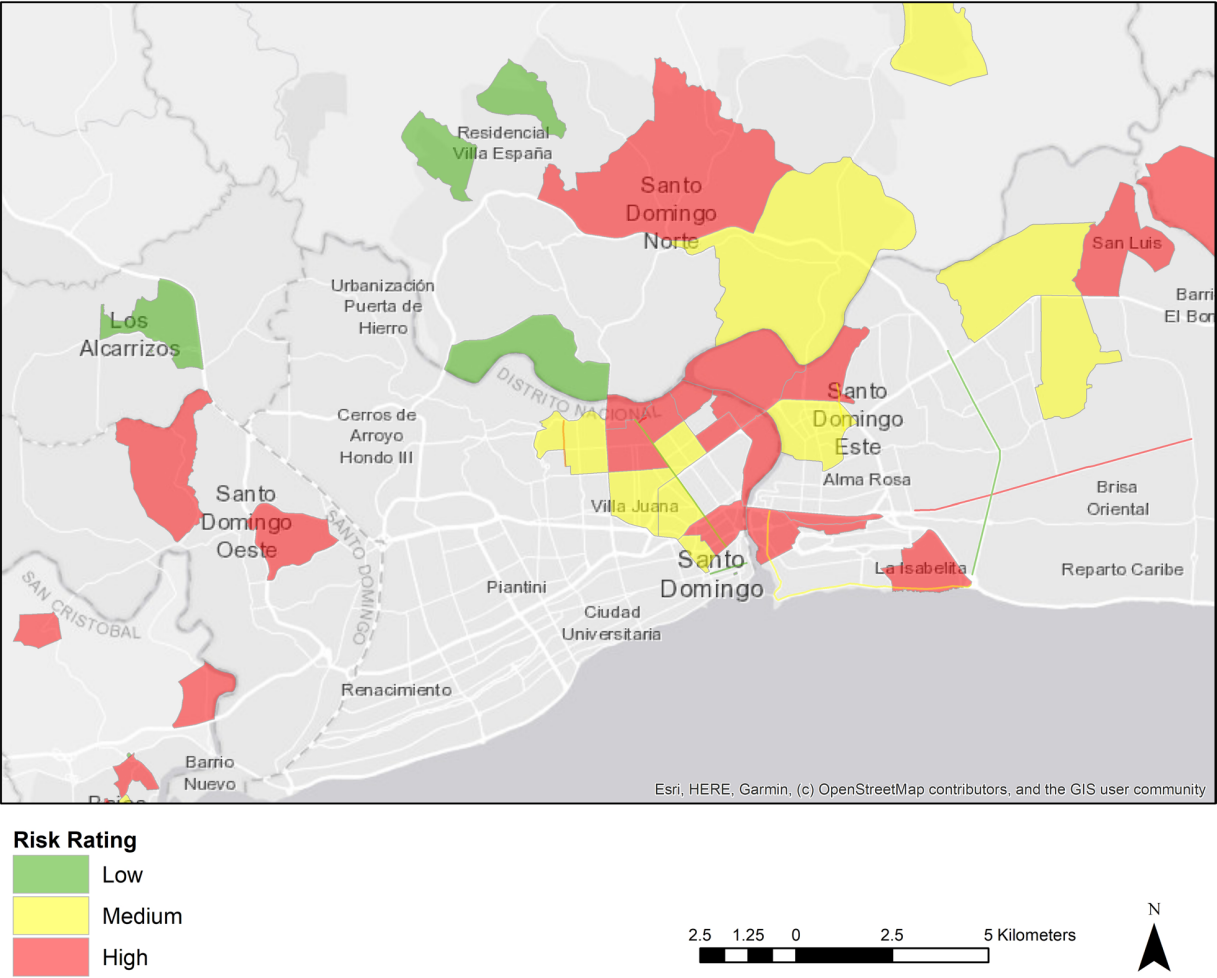
Map of FSW perceived risk environments in Santo Domingo by classification of risk, 2019

Descriptive statistics from the participatory geographic mapping exercise of perceived risk environments are presented in Table 1. Participants listed 62 neighborhoods in Santo Domingo as risk areas. Among the neighborhoods listed, 77% were classified as unsafe areas due to the presence of drugs. Sixty percent were classified as risk areas due to high rates of crime and violence, and 47% were described as risk areas due to high levels of poverty. Ninety-three unique establishments were identified as risk hot spots. These included specific hotels, parks, markets, bars/discos, *colmados* (corner stores that sell alcohol and are frequent gathering spots), drug markets, sex work venues, and street intersections. The majority of these establishments (85%) were described as locations where sex work could be solicited or exchanged. Eight streets were reported as risk locations. The streets were classified as locations with heavy police presence and where sex workers could be found. Due to the sensitivity of this information and concerns of safety, the names and exact geographic location of the establishments and streets and will not be published. Figure 3 is a choropleth map of the larger geographic areas reported by participants as risk environments, where red equals high risk, yellow equals medium risk and green equals low risk.

**Table 1:** Identified categories of risk spaces and characteristics in Santo Domingo from the perspective of FSWs living with HIV (N = 51)

Perceived risk environments	Risk characteristics	Percent of perceived risk environments with the identified risk characteristic
Neighborhoods (N = 62)	Drugs (use, selling, trafficking)	77.42%
	Violence and crime	59.68%
	Heavy policing	35.48%
	Sex work	19.35%
	Poverty	46.77%
Specific establishments (N = 93)^a^		
	Drugs (use, selling, trafficking)	26.88%
	Violence and crime	8.60%
	Heavy Policing	8.60%
	Sex Work	84.95%
	Poverty	1.08%
Common streets (N = 8)^b^		
	Drugs (use, selling, trafficking)	50.00%
	Violence and crime	25.00%
	Heavy policing	100.00%
	Sex work	87.50%
	Poverty	25.00%

^a^Establishments included hotels, bars/discos, *colmados* (corner stores that sell alcohol and are frequent gathering spaces), markets, parks, street intersections, drug markets, and sex work venues as listed by participants

^b^Common streets included highways and central avenues

Notes. *FSWs* female sex workers; % based on non-missing data (< 10% missing on any variable)

### Daily activity space travel diary

**Fig. 4:**
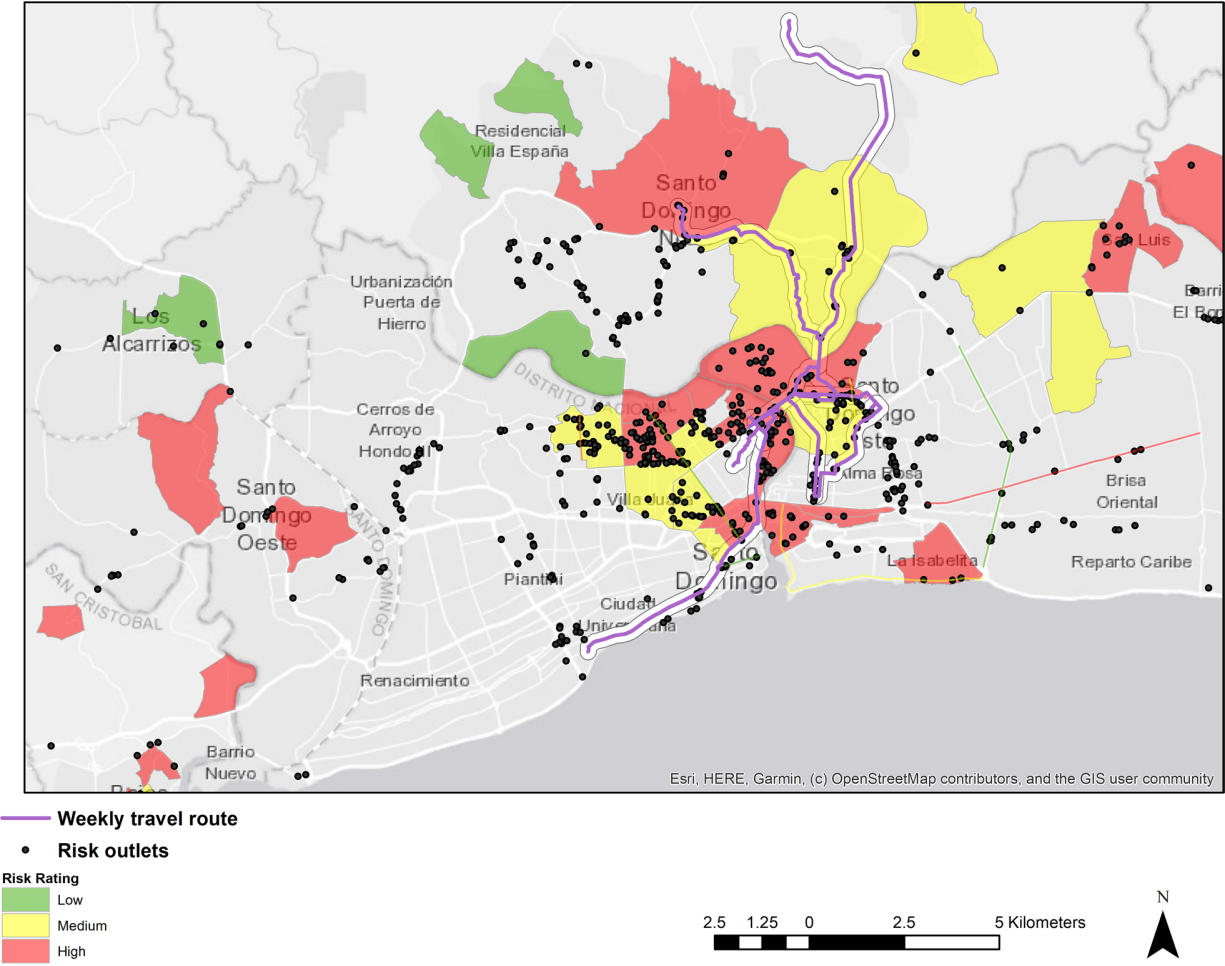
Map of one participant’s weekly activity path overlaid on a base map of Santo Domingo, FSW perceived risk environments, and 2014 PLACE study risk outlet data, 2019

Table 2 provides basic summary statistics for participant daily activity paths. A total of 311 of 357 travel diaries were submitted on time equating to a response rate of 87% and the average number of diaries completed on time was 6 with a minimum of 0 and a maximum of 7. A total of 1740 points were recorded over the one-week data collection period. The approximated average time tracked per day was 1175 [range 30–1440] minutes and the approximated average daily distance traveled was 17,158 [range 0–395569] meters. The mean number of activity locations per day was 4 [range 1–11]. Figure 4 provides a visual representation of one participant’s weekly activity path overlaid on a base map of Santo Domingo risk areas and risk outlet data from the 2014 PLACE study.

**Table 2:** Activity space characteristics among FSWs living with HIV in Santo Domingo (N = 51 participants, 1740 points recorded and 339 observations over 7 days)

	Mean	SD	Range
Average number of travel diaries completed	6	0.9	[0–7]
Average number of locations visited per day	4	1.5	[1–11]
(Approximate) average time tracked per day (minute)	1175	365.1	[30–1440]
(Approximate) average path distance per day (meters)	17,158	29,022.0	[0–395569]

Notes. *FSWs* female sex workers, *SD* standard deviation; mean based on non-missing data (< 10% missing on any variable)

## Discussion

This pilot study was among the first to demonstrate the feasibility of measuring risk environments of a key population in a LMIC. In areas where neighborhood-level data may be sparce, techniques such as participatory geographic mapping and ecological activity space assessments through a travel diary, coupled with participant surveys may be utilized to capture and quantify risk environment exposures.

### Participatory geographic mapping of perceived risk environments

In the Dominican Republic, neighborhood-level data is sparse. Shapefiles for administrative boundaries and roads are available from the National Office of Statistics (ONE), but descriptive data at the lowest administrative boundary (*barrio paraje*) are limited. to population, household education, female-headed households, and mean household socio-economic status. Thus, to obtain information on risk environments in Santo Domingo we used participatory geographic mapping. Using Google Maps, we worked with participants to identify and characterize locations of perceived risk in the city. Participants enjoyed the interactive aspect of the participatory geographic mapping activity. Visually identifying risk locations and seeing emerging risk “hot spots” helped them conceptualize the idea of a risk environment and how spending time in those places could potentially influence behaviors. Google Maps is a free mapping tool that is easy to use. We were able to directly save each participant’s risk environment map and export the corresponding data on locations and geographic coordinates into Excel and eventually ArcGIS 10.6 (ESRI, Redlands, CA).

The most common barrier with the implementation of this approach was the difficulty in locating a point or area due to limited and outdated satellite imagery of Santo Domingo on Google Maps. Another issue was that some participants were not accustomed to reading a map or giving directions. To address these difficulties, we used Google Street View and worked with the participant to identify landmarks, major streets, and geographic areas in the approximate area of the location being mapped. Although the method of participatory geographic mapping is subjective and only provided risk data for some enumeration areas, consistent patterns did emerge which was reassuring for data reliability.

### Daily activity space travel diary

Few studies have explored the experiences of FSWs in socio-geographical contexts outside defined administrative boundaries (i.e., neighborhoods, work environments). Neighborhood and built environment studies among FSWs typically use the sex work venue as the geographic “unit” of analysis [5]. However, considering the substantial increase in non-establishment-based sex work and the pervasive societal stigma attached to sex work, the venue may not be the most relevant space influencing FSWs decision-making processes and associated behaviors. Furthermore, with the aid of social media and text-based mobile platforms, the notion of a “fixed” sex work environment has evolved.

To better understand participants’ daily exposure to risk environments we used the novel approach of activity space mapping. Documenting and analyzing activity spaces of FSWs provided a detailed picture of the social and spatial risk environments in their daily lives, and how such contextual exposures may contribute to risk behaviors. FSWs daily activity patterns traversed many areas, exposing them to multiple social and physical contexts and experiences.

To track participant’s daily routes over the course of one week we used a paper travel diary where participants recorded where they went each day. We used incentive-based completion for the travel diary where compensation increased per item completed. This approach appeared to have a positive effect and minimized respondent drop-out. Eighty-seven percent of participants submitted the travel diaries on time during the week, meaning that they submitted the WhatsApp photo of the completed travel dairy by 12:00 PM of the following day. All participants returned at the end of the 7-day data collection period and no travel diaries were lost. The use of WhatsApp to send a photo of the completed travel diary worked well. Only 1 participant did not have access to a smartphone and she was also illiterate. In this case, the participant called the PI every evening to report on her daily travel and activities.

In exit interviews with study participants, women reported that they enjoyed the travel diary. They stated that the travel diary gave them more awareness of how much they moved over the course of the day and that it made them more attentive to the spaces they frequented. Some participants struggled with writing and recording the exact address of each location as they were not accustomed to looking at street names. The use of symbols was an effective way to safely capture information related to illicit activities and to minimize writing for participants. All participants reported that now that they had a better understanding of activity space mapping, in future studies, they would be open to using a GPS tracking device to record their daily travel paths. However, the use of GPS devices requires that protective measures such as de-identification of location data, secure storage of data, password protected computers, restricted access to data to one team member, and spatial confidentiality when publishing, are put in place to address ethical issues and the safety of the study population. Finally, it is critical that the study population is clear on the data that the device is collecting and feel comfortable carrying the device for the duration of the study.

## Strengths and limitations

This study is not without limitations. First, as seen in Figs. 2 and 3, many geographic areas are blank because they were not mentioned as perceived risk areas during the participatory geographic mapping exercise**.** In turn, for participants whose daily activity paths cross geographic spaces with no data, exposure estimates will be biased. Second, it is important to consider that characteristics of geographic spaces are not stagnant nor are perceptions of place. A place may be defined as high risk at one moment but be considered low risk at another. Such changes may be attributable to actual physical changes in the environment or one’s own change of perception of risk classifications. To document these changes, study designs could consider participatory geographic mapping exercises over-time. Third, given the stigma associated with HIV, sex work, and drug use in the Dominican Republic, it is possible that participants did not reveal true locations of risk. Validated measurements and multiple modes of data collection were used to minimize information bias. Fourth, because daily activity space data were not collected using GPS devices, but based on participants reporting of the address and then locating the address on Google Maps, spatial imprecision and error are likely present. Time spent at each location is subject to recall and reporting bias. Finally, study participants were financially compensated for study participation. While this could have influenced response rates, we worked with the local study team and IRB to ensure incentive amounts were not coercive and aligned with the going rate in country and participant time invested.

Despite limitations, this study has several strengths. The methods presented in this study demonstrate application of spatial epidemiology in a LMIC. The participatory geographic mapping approach helped mitigate challenges inherent to limited spatial data in such contexts and among vulnerable populations. The use of daily travel diaries was an innovative approach to collect activity space data in order to calculate more precise operationalized measures of risk exposure. These methods may be generalizable to other high-risk populations and may have broader application for informing health interventions. One goal of this pilot study was to test the methods among the population and to evaluate feasibility and acceptability. Because we used formative research to help refine the data collection tools and consulted with potential participants and the study team, the methods were acceptable to the specific study population. To increase efficiency for a larger study, more financial resources would be beneficial in order to hire and train more interviewers, to purchase more computers, and to provide compensation to participants. Interviewers need to be trained on how to use Google Maps, should have a good understanding and knowledge of the city where the study is being conducted, understand the participatory component of the mapping exercise, and also be able to demonstrate how to complete the travel diary. Furthermore, strong skills in data management are important for tracking daily completion of the travel diaries. Finally, the study adds to the limited body of place-based research in an international setting. There has been little research in the Dominican Republic that has used socio-spatial methods to examine perceived risk environments and exposure of key populations, especially those already infected with HIV.

## Conclusion

The methods presented in this study were successful in generating contextual data to assess the role of daily risk environments on risk behaviors among FSWs living with HIV in a LMIC. The methods described can generate place-based data in settings where access to such data may be limited. Furthermore, the methods were interactive and educational, allowing study participants to take an active role in the data collection process and providing insight into how exposure to different environments may influence their behaviors.

## Data Availability

The datasets generated and/or analyzed during the current study are not publicly available due to the sensitivity of the population under study and potential harm that could arise from sharing their information. Data are available from the corresponding author on reasonable request.

## References

[1] Rhodes T, Singer M, Bourgois P (2005). The social structural production of HIV risk among injecting drug users. Soc Sci Med.

[2] Rhodes T (2009). Risk environments and drug harms: A social science for harm reduction approach. Int J Drug Policy.

[3] Brouwer KC, Weeks JR, Lozada R, Thomas YF, Richardson D, Cheung I (2008). Integrating GIS into the study of contextual factors affecting injection drug use along the Mexico/US border. Geography and drug addiction.

[4] Diez Roux AV (2001). Investigating neighborhood and area effects on health. Am J Public Health.

[5] Conners EE, West BS, Roth AM (2016). Quantitative, qualitative and geospatial methods to characterize HIV risk environments. PLoS ONE.

[6] Kawachi I, Berkman L (2003). Neighborhoods and health.

[7] Messer L, Kaufman J, Dole N (2006). Violent crime exposure classfication and adverse birth outcomes: a geographically-defined cohort study. Int J Health Geogr.

[8] Clark A, Scott D (2013). Understanding the impact of the modificable areal unit problem on the relationship between active travel and the built environment. Urban Stud.

[9] Kwan M-P (2012). The uncertain geographic context problem. Ann Am Assoc Geogr.

[10] Matthews SA, Yang TC (2013). Spatial polygamy and contextual exposures (SPACES): promoting activity space approaches in research on place and health. Am Behav Sci.

[11] Brouwer KC, Rusch ML, Weeks JR (2012). Spatial epidemiology of HIV among injection drug users in Tijuana, Mexico. Ann Am Assoc Geogr.

[12] Ansumana R, Malanoski A, Bockarie A (2010). Enabiling methods for community health mapping in developing countries. Int J Health Geogr.

[13] Verplanke J, McCall M, Uberhuaga C (2016). A shared perspective for PGIS and VGI. Cartogr J.

[14] Smith K, Barret C, Box P (2000). Participatory risk mapping for targeting research and assistance: with an example from East African pastoralist. World Dev.

[15] Goldenberg S, Deering K, Amram O (2017). Community mapping of sex work criminalization and violence: impacts on HIV treatment interruptions among marginalized women living with HIV in Vancouver, Canada. Int J STD AIDS.

[16] Livingston K, Padilla M, Scott D, et al. Methods of mapping ethnographic data on migration, tourism labor, and health risk in the Dominican Republic. Fla Geogr. 2016;47. PMC5028012 27656039

[17] Kwan M-P (2013). Beyond space (as we knew it): toward temporally integrated geographies of segregation, health, and accessibility. Ann Am Assoc Geogr.

[18] Duncan DT, Regan SD (2016). Mapping multi-day GPS data: a cartographic study in NYC. J Maps.

[19] Duncan DT, Kapadia F, Regan SD (2016). Feasibility and acceptability of global positioning system (GPS) methods to study the spatial contexts of substance use and sexual risk behaviors among young men who have sex with men in New York City: A P18 cohort sub-study. PLoS ONE.

[20] Byrnes HF, Miller BA, Wiebe DJ (2015). Tracking adolescents with global positioning system-enabled cell phones to study contextual exposures and alcohol and marijuana use: a pilot study. J Adolesc Health.

[21] Vaughan AS, Kramer MR, Cooper HL (2017). Activity spaces of men who have sex with men: an initial exploration of geographic variation in locations of routine, potential sexual risk, and prevention behaviors. Soc Sci Med.

[22] Rojas P, Malow R, Ruffin B (2011). The HIV/AIDS epidemic in the Dominican Republic: key contributing factors. J Int Assoc Physicians AIDS Care (Chic).

[23] Carrasco MA, Nguyen TQ, Barrington C (2018). HIV stigma mediates the association between social cohesion and consistent condom use among female sex workers living with HIV in the Dominican Republic. Arch Sex Behav.

[24] Carrasco MA, Barrington C, Kennedy C (2017). ‘We talk, we do not have shame’: addressing stigma by reconstructing identity through enhanicng social cohesion among female sex workers living with HIV in the Dominican Republic. Cult Health Sex.

[25] Kerrigan D (2016). Stigma, cohesion, and HIV outcomes among vulnerable women across epidemic settings.

[26] Chorowodza A, van Rooyen H, Joseph P (2009). Using participatory methods and geographic information systems (GIS) to prepare for an HIV community-based trial in Vulindlela, South Africa (Project Accept- HPTN 043). J Community Psychol.

[27] Strathdee S, West B, Reed E (2015). Substance use and HIV among female sex workers and female prisoners: risk environmnets and implications for prevention, treatment, and policies. J Acquir Immune Defic Syndr.

[28] Sampson RJ, Raudenbush SW, Earls F (1997). Neighborhoods and violent crime: a multilvel study of collective efficacy. Science.

[29] USAID (2014). Prioridades para los esfuerzos locale de control de VIH (PLACE) en la Republica Dominicana.

